# Supplementary material for: Anterior cingulate cortex and its input to the basolateral amygdala control innate fear response
Source: Nat Commun. 2018 Jul 16;9:2744. doi: 10.1038/s41467-018-05090-y (PMC6048069; doi:10.1038/s41467-018-05090-y)
Supplement: Supplementary file 1 — Supplementary Information [file 41467_2018_5090_MOESM1_ESM.pdf]

Supplementary Material for

Anterior Cingulate Cortex and its Input to the Basolateral Amygdala Control Innate Fear Response

Jhang et al.

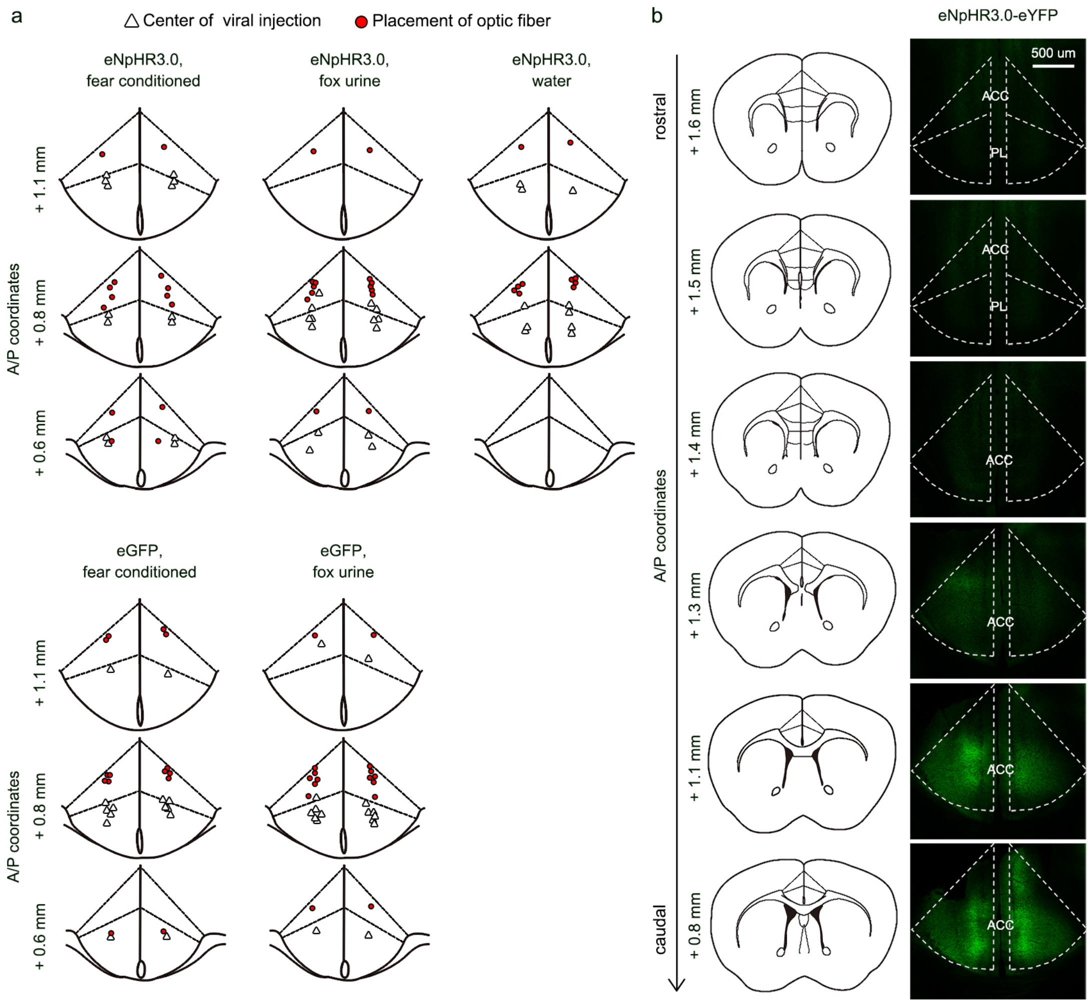

Supplementary Figure 1

Histological confirmation of the caudal ACC-targeted photoinhibition

a, Schematic images indicating injection center and fiber-tip placement used for photoinhibition of the ACC somata. Each column of diagram is related to Fig. 1d, 1j, 1n, 1e, and 1k. Target coordinate is A/P +0.8 mm, M/L ±0.35 mm, D/V -1.8 (injection) or D/V -1.5 mm (optic fiber) from bregma. The triangles and red dots represent each instance of injection center and fiber placement, respectively.

b, Consecutive epi-fluorescence images showing the selective expression of eNpHR3.0-eYFP in the caudal ACC (representative case from one mouse). Scale bar, 500 µm

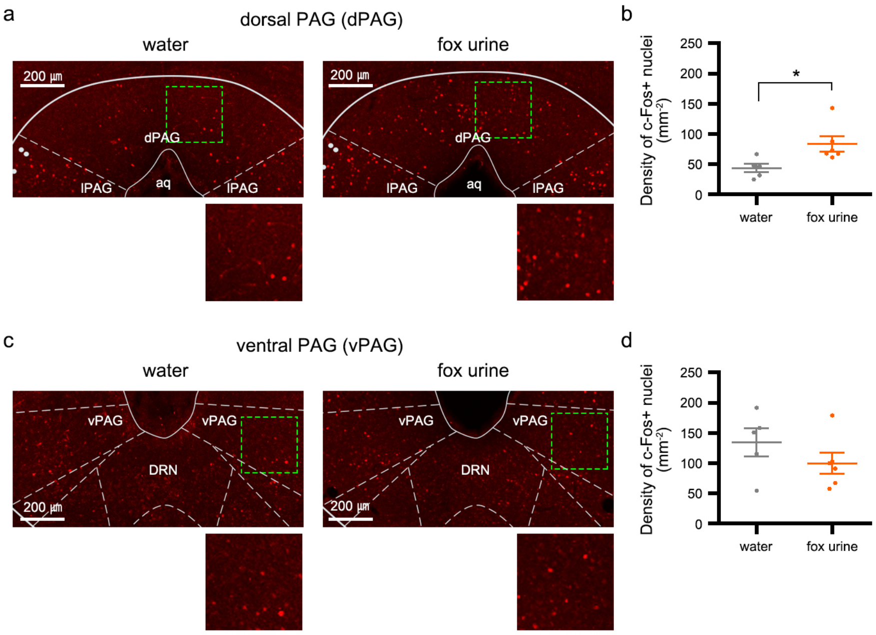

Supplementary Figure 2

Increase of c-Fos+ cells in the dPAG (but not vPAG) after exposure to fox urine

a, Representative confocal images showing the c-Fos+ nuclei observed in the dPAG in water (left) and fox urine (right) groups. Bottom images are magnification of the green dashed area.

b, Density of c-Fos+ nuclei observed in the dPAG measured in water (n = 5 mice) and fox urine (n = 6 mice) exposed groups. Unpaired *t*-test, ($P = 0.0283$, $t = 2.609$, df = 9).

c, Representative confocal images showing the c-Fos+ nuclei observed in the vPAG, in water (left) and fox urine (right) groups. Bottom images are magnification of the green dashed area.

d, Density of c-Fos+ nuclei observed in the vPAG measured in water (n = 5 mice) and fox urine (n = 6 mice) exposed groups. Unpaired *t*-test, ($P = 0.2597$, $t = 1.203$, df = 9).

aq, aqueduct; dPAG, dorsal periaqueductal gray; lPAG, lateral periaqueductal gray; vPAG, ventral periaqueductal gray; DRN, dorsal raphe nucleus. Scale bar, 200 μm.

Data are expressed as mean ± s.e.m. $*P < 0.05$

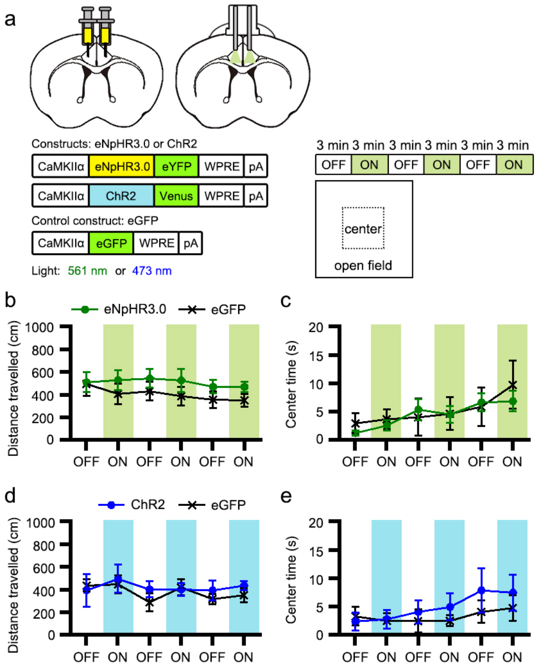

Supplementary Figure 3

Manipulation of CaMKIIα-positive neurons in the ACC does not affect locomotor and anxiety behaviors in open field test

a, Illustration of open field test with optogenetic manipulation of CaMKIIα-positive neurons in the ACC.

b, **c**, Distance travelled (**b**) and time in center (**c**) are not affected by the light delivery in eNpHR3.0 group (n = 8) compared to eGFP control group (n = 8). (**b**) Repeated measures two-way ANOVA ($F_{(5,70)}$ = 0.2112, P = 0.9567) and Sidak's post hoc test. (**c**) Repeated measures two-way ANOVA ($F_{(5,70)}$ = 0.5755, P = 0.7185) and Sidak's post hoc test.

d, **e**, Distance travelled (**d**) and time in center (**e**) are not affected by the light delivery in ChR2 group (n = 7) compared to eGFP control group (n = 8). (**d**) Repeated measures two-way ANOVA ($F_{(5,65)}$ = 0.4548, P = 0.8083) and Sidak's post hoc test. (**e**) Repeated measures two-way ANOVA ($F_{(5,70)}$ = 0.5161, P = 0.7631) and Sidak's post hoc test.

Data are expressed as mean ± s.e.m.

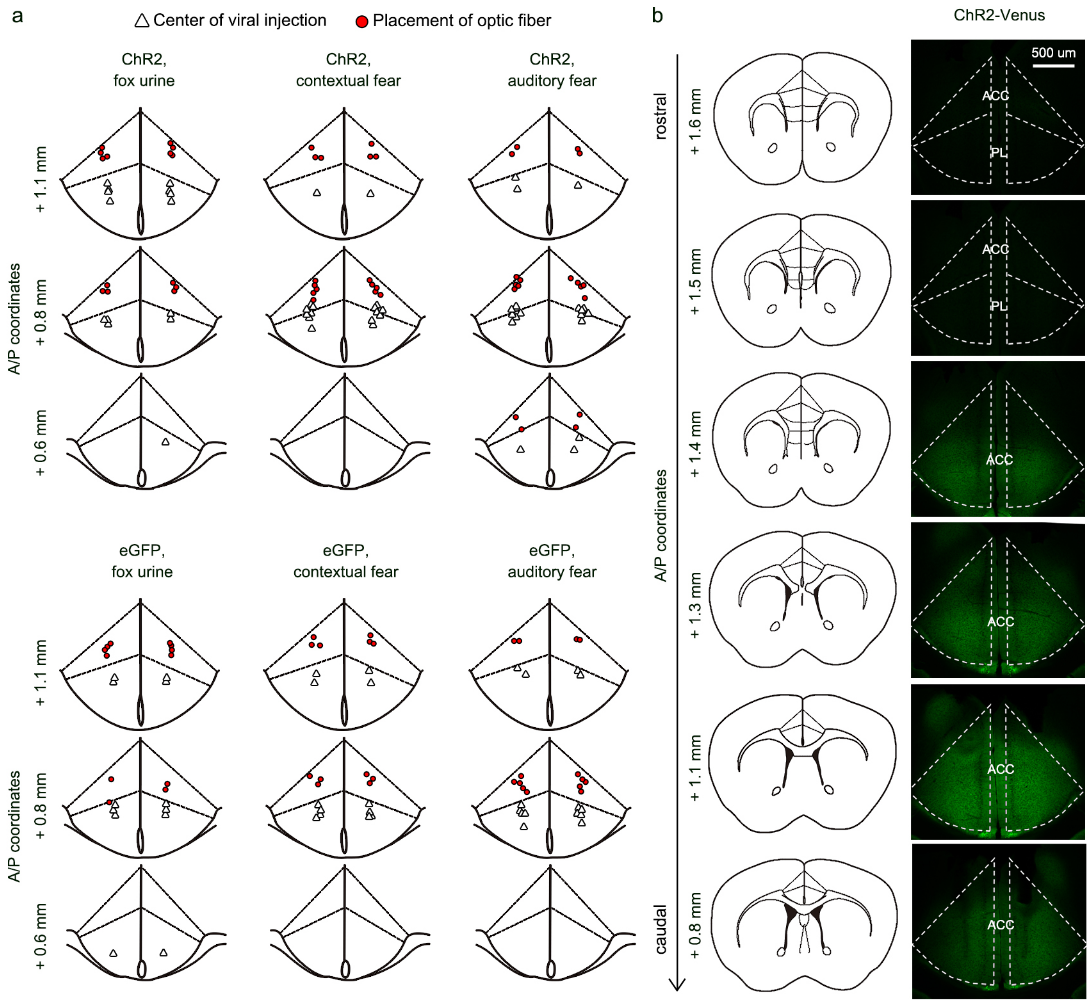

Supplementary Figure 4

Histological confirmation of the caudal ACC-targeted photoactivation

a, Schematic images indicating injection center and fiber-tip placement used for photoactivation of the ACC somata. Each column of diagram is related to Fig. 2c, 3c, 3g, 2d, 3d, and 3h. Target coordinate is A/P +0.8 mm, M/L ±0.35 mm, D/V -1.8 (injection) or D/V -1.5 mm (optic fiber) from bregma. The triangles and red dots represent each instance of injection center and fiber placement, respectively.

b, Consecutive epi-fluorescence images showing the selective expression of ChR2-Venus in the caudal ACC (representative case from one mouse). Scale bar, 500 µm

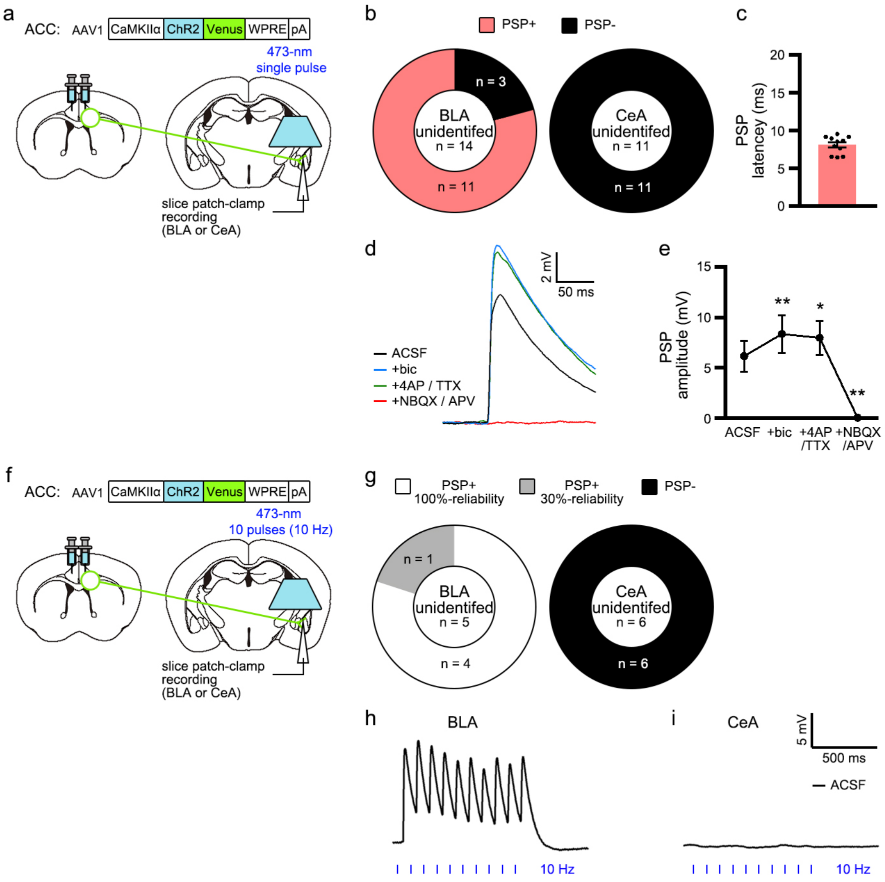

Supplementary Figure 5

Ex vivo patch-clamp recording of BLA neurons' response to photostimulation of ACC terminals

a, Illustration of ex vivo patch-clamp recording strategy for monitoring light-evoked PSPs from input-unidentified BLA neurons.

b, Proportion of BLA or CeA neurons showing PSP response to light stimulation. Total 14 cells (from 14 slices of 5 mice) were recorded, and 11 cells displayed PSP response. None of CeA cells (selected in the same slices) displayed PSPs.

c, Representative recording traces for light-induced PSPs. Responses were monitored with the serial treatments of ACSF (black), +bicuculline (blue), bicuculline +TTX +4-AP (green), and bicuculline +TTX +4-AP +NBQX +APV (red).

d, Latency of light-induced PSP response. (n = 11, PSP+ cells in the BLA).

e, Summary of amplitudes of light-evoked PSPs measured from each drug treatment condition (n = 11 cells from 14 slices of 5 mice). Repeated measures one-way ANOVA ($F_{(1.138,\ 11.38)}$ = 19.30, P = 0.0008) and Dunnett's post hoc test (ACSF vs. +Bic, P = 0.0021; vs. +4-AP/TTX, P = 0.0150; vs. +NBQX/APV, P = 0.0057). Data are expressed as mean ± s.e.m. *P < 0.05, **P < 0.01

f, Illustration of ex vivo patch-clamp recording strategy for monitoring the PSP reliability to 10-Hz stimulation. Recording was performed in ACSF condition. Light power was approximately matched to in vivo-like conditions used for behavior experiments.

g, Proportion of BLA and CeA neurons showing PSP response to light stimulation. 4 of 5 randomly-selected BLA neurons displayed 100% reliability. One BLA neuron displayed 30% reliability, and none of CeA neurons displayed PSP response (PSP-).

h, **i**, Representative recording traces for induced PSP responses to 1-s of 10-Hz light stimulation

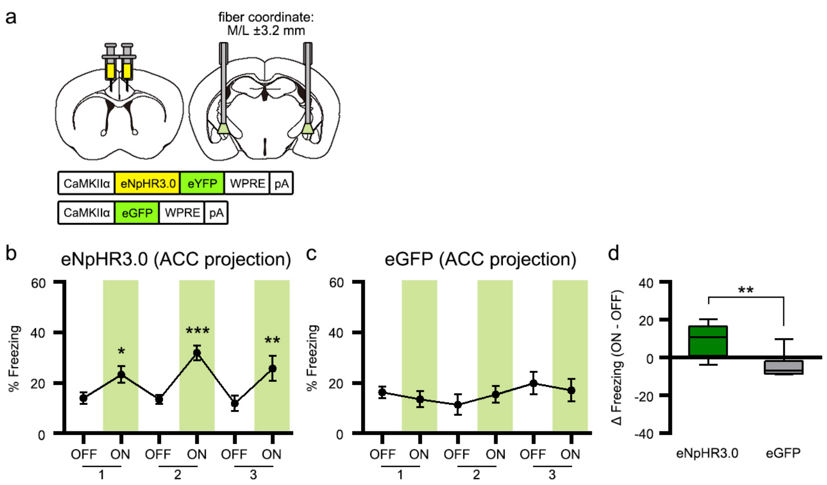

Supplementary Figure 6

Photoinhibition of ACC-amygdala projection increases the freezing response to fox urine

a, Illustration of the eNpHR3.0-based inhibition of ACC projection in the amygdala.

b, **c**, Percentage of time spent freezing during the fox urine test was selectively increased by photoinhibition in the eNpHR3.0 group (n = 8) (**b**) but not in the eYFP group (n = 8) (**c**). Paired *t*-tests for eNpHR3.0 (*P* = 0.0158, < 0.0001, = 0.0074) or Wilcoxon matched-pair rank tests for eGFP (*P* = 0.1953, = 0.2500, = 0.3828).

d, Light-induced change in percentage of freezing behavior calculated by subtracting freezing values within the first OFF-ON cycle for eNpHR3.0 (**b**) and eGFP (**c**) groups. Mann-Whitney U test (*P* = 0.0030).

Line graphs are expressed as mean ± s.e.m. Box-whisker plot is expressed as median, interquartile range with 5-95 percentile distribution. *P < 0.05, **P < 0.01, ***P < 0.001

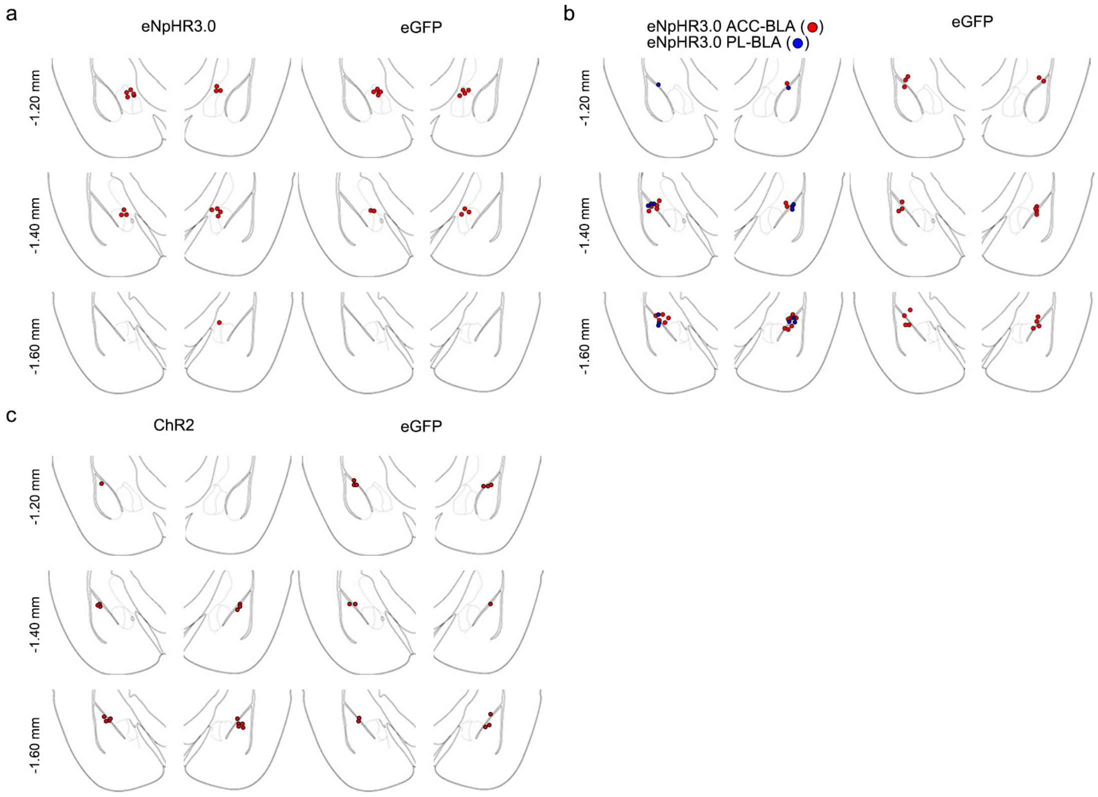

Supplementary Figure 7

Placement of optic fiber for bidirectional optogenetic manipulation of ACC terminals in the amygdala

a, Schematic illustration of fiber-tip placement used for photoinhibition of ACC projection terminals. Target coordinate is A/P -1.6 mm, M/L ±3.2 mm, D/V -4.5 mm from bregma.

b, Schematic illustration of fiber-tip placement used for photoinhibition of ACC or PL projection terminals. Target coordinate is A/P -1.6 mm, M/L ±3.35 mm, D/V -4.5 mm from bregma.

c, Schematic illustration of fiber-tip placement used for photoactivation of the ACC projection terminals. Target coordinate is A/P -1.6 mm, M/L ±3.35 mm, D/V -4.5 mm from bregma.

The red or blue dots denote each instance of fiber-tip placement

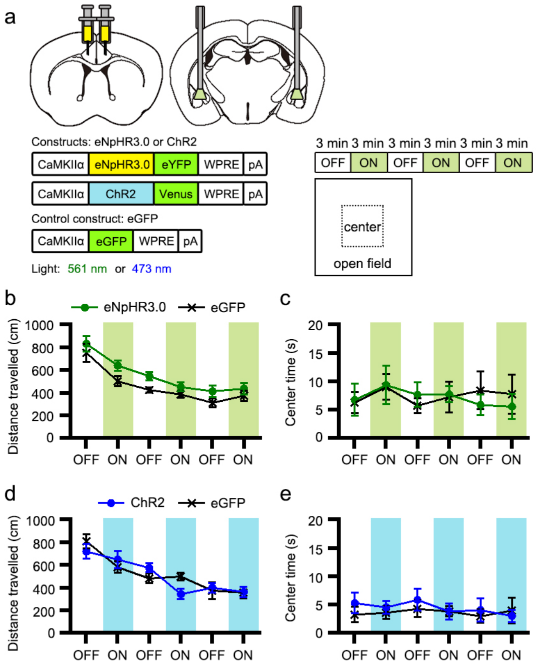

Supplementary Figure 8

Manipulation of the ACC-BLA projection input does not affect locomotor and anxiety behaviors in open field test

a, Illustration of open field test with optogenetic manipulation of ACC-BLA projection.

b, **c**, Distance travelled (**b**) and time in center (**c**) were not affected by the light delivery in eNpHR3.0 group (n = 10) compared to eGFP control group (n = 10). (**b**) Repeated measures two-way ANOVA ($F_{(5,90)}$ = 0.2604, P = 0.9335) and Sidak's post hoc test. (**c**) Repeated measures two-way ANOVA ($F_{(5,70)}$ = 0.4473, P = 0.8149) and Sidak's post hoc test.

d, **e**, Distance travelled (**d**) and time in center (**e**) are not affected by the light delivery in ChR2 group (n = 8) compared to eGFP control group (n = 7). (**d**) Repeated measures two-way ANOVA ($F_{(5,65)}$ = 2.199, P = 0.0649) and Sidak's post hoc test. (**e**) Repeated measures two-way ANOVA ($F_{(5,65)}$ = 0.5698, P = 0.7228) and Sidak's post hoc test.

Data are expressed as mean ± s.e.m.

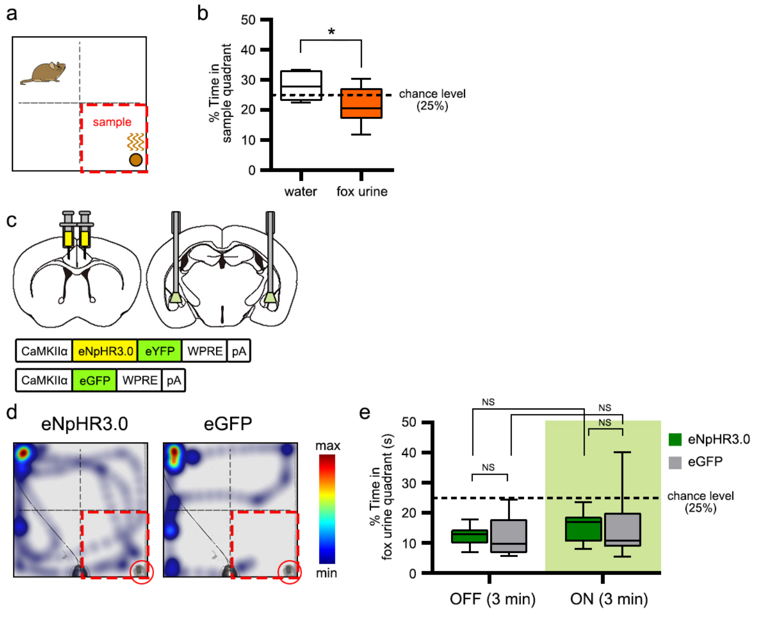

Supplementary Figure 9

Photoinhibition of ACC-BLA projection does not alter avoidance response to fox urine

a, Illustration of the fox urine avoidance test.

b, Percentage of time spent in the sample quadrant. Mice in fox urine group (n = 8) spent less time (%) in the sample quadrant compared to water control group (n = 8). Unpaired *t*-test (P = 0.0319, t = 2.383, df = 14).

c, Illustration of the eNpHR3.0-based photoinhibition of the ACC- BLA projection.

d, Representative heat map images showing the representative time-spending pattern of eNpHR3.0- or eGFP-expressing mice during the initial 3 min (light OFF) of avoidance test. Red dashed square represents the sample quadrant. Red circle denotes the location of sample dish.

e, Percentage of time spent in the sample quadrant during 3-min OFF and 3-min ON periods, of eNpHR3.0 (n = 9) and eGFP (n = 7) groups. Repeated measures two-way ANOVA ($F_{(1, 14)}$ = 0.0505, P = 0.8254) with Sidak's post hoc test.

Box-whisker plots are expressed as median, interquartile range with 5-95 percentile distribution. *P < 0.05; NS, not significant (P > 0.05)
